# Efficacy and potential mechanisms of Chinese herbal compounds in coronavirus disease 2019: advances of laboratory and clinical studies

**DOI:** 10.1186/s13020-021-00542-y

**Published:** 2021-12-03

**Authors:** Mu-Feng Xiang, Cheng-Tao Jin, Li-Hua Sun, Zhi-Hui Zhang, Jing-Jing Yao, Liu-Cheng Li

**Affiliations:** 1 Department of Pharmacy, Sir Run Run Shaw Hospital, School of Medicine, Zhejiang University, Hangzhou, 310016 China; 2 Department of Diagnostic Ultrasound & Echocardiography, Sir Run Run Shaw Hospital, School of Medicine, Zhejiang University, Hangzhou, China; 3 Institute of Vascular Disease, Shanghai TCM-Integrated Hospital, Shanghai University of Traditional Chinese Medicine, Shanghai, 200082 China; 4 Key Laboratory of Industrial Dust Prevention and Control & Occupational Health and Safety, Ministry of Education, Anhui University of Science and Technology, Huainan, 232001 China

**Keywords:** Coronavirus, COVID-19, SARS-CoV-2, Chinese herbal compounds

## Abstract

The Coronavirus disease 2019 (COVID-19) pandemic is still spread and has made a severe public health threat around the world. To improve disease progression, emerging Chinese herbal compounds were used in clinical practice and some agents have proven beneficial in treating COVID-19. Here, the relevant literature from basic researches to clinical application were identified and comprehensively assessed. A variety of Chinese herbal compounds have been reported to be effective in improving symptoms and outcomes in patients with COVID-19, particularly together with routine treatment strategy. The pharmacological activities were mainly attributed to the relief of clinical symptoms, inhibition of cytokine storm, and improvement of organ function. Besides, the development of novel antiviral drugs from medicinal herbs were further discussed. The updated laboratory and clinical studies provided the evidence of Chinese herbal compounds such as Lianhua Qingwen prescription, Shufeng Jiedu prescription, and Qingfei Paidu Tang for the relief of COVID-19. However, both of the randomized controlled trials and real world researches need to be done for supporting the evidence including the efficacy and safety in fighting COVID-19.

## Introduction

Respiratory disease is one of leading cause of death across the globe and bring great challenges to human health [1–3]. Coronavirus disease 2019 (COVID-19), infected with Severe Acute Respiratory Syndrome (SARS) Coronavirus 2 (SARS-CoV-2), has led to more than 250 million infections and 5 million deaths till now [4]. The World Health Organization (WHO) announced that COVID-19 became a global pandemic in March 2020.

COVID-19 was characterized by varied clinical symptoms including fever, cough, and fatigue, among whom the severe cases would result in acute respiratory distress syndrome (ARDS), as well as multiple organ failure, even death [5–7]. It remains grim to contain the spread and explore its effective treatment strategies. Since the early outbreak of COVID-19, China took timely measures to limit its spread, and officially issued several versions of the Guideline on Diagnosis and Treatment of Coronavirus Disease 2019. Traditional Chinese medicine (TCM) was recommended for the clinical use in China [5].

TCM have been used for thousands of years to prevent and cure various diseases, and have been a part of the integrative medicine field for developing novel drugs [8–10]. Fighting against SARS-CoV-2 infection provides an opportunity to test the true value of TCM in treating emerging contagious diseases. It is encouraging that recent data had proved that the oral preparations of Chinese herbal compounds were important agents for treating COVID-19 with symptom relief, virus inhibition, and immune function improvement, especially combined with conventional treatment regimens likely western medicine. This review will focus on providing an insight into the advances of current experimental and clinical experience of Chinese herbal compounds in treating COVID-19 (Table 1), to offer timely data delivery in human medicine.

**Table 1:** The herbal sources of Chinese herbal compounds

Agents	Herbal sources	References
Lianhua Qingwen prescription	*Forsythia suspensa* (Thunb.) Vahl. (Lianqiao), *Lonicera japonica* Thunb. (Jinyinhua), *Ephedra sinica* Stapf (Mahuang), *Armeniacae Amarum* Semen (Kuxingren), *Gypsum Fibrosuum* (Shigao), *Isatis tinctoria* L. (Banlangen), *Dryopteris crassirhizoma* Nakai (Mianmaguanzhong), *Houttuynia cordata* Thunb. (Yuxingcao), *Pogostemon cablin* (Blanco) Benth. (Guanghuoxiang), *Rheum palmatum* L. (Dahuang), *Rhodiola rosea* Linn. (Hongjingtian), *Mentha haplocalyx* Briq. (Bohe), *Glycyrrhiza uralensis* Fisch. (Gancao)	[5, 12, 17]
Shufeng Jiedu prescription	*Reynoutria japonica* Houtt. (Huzhang), *Forsythia suspensa* (Thunb.) Vahl. (Lianqiao), *Isatis tinctoria* L. (Banlangen), *Bupleurum chinensis* DC. (Chaihu), *Patrinia villosa* Juss. (Baijiangcao), *Verbena officinalis* Linn. (Mabiancao), *Phragmites communis* Trin. (Lugen), *Glycyrrhiza uralensis* Fisch. (Gancao)	[24, 28–30]
Huoxiang Zhengqi formulation	*Atractylodes Lancea* (Thunb.) DC. (Cangzhu), *Pericarpium Citri* Reticulatae (Chenpi), *Magnolia officinalis* Rehd. et Wils. (Houpu), *Angelica dahurica* Fisch. ex Hoffm. Benth. et Hook.f. (Baizhi), *Poria cocos* (Schw.) Wolf. (Fuling), *Areca catechu* L. (Dafupi), *Pinellia ternata* (Thunb.) Breit. (Banxia), *Glycyrrhiza uralensis* Fisch. (Gancao), *Pogostemon cablin* (Blanco) Benth. (Guanghuoxiang), *Perilla frutescens* (L.) Britt.var. crispa (Thunb.) Hand.-Mazz. (Zisuye)	[35, 36]
Qingfei Paidu Tang	*Ephedra sinica* Stapf (Mahuang), *Glycyrrhiza uralensis* Fisch. (Gancao), *Armeniacae Amarum* Semen (Kuxingren), *Gypsum Fibrosuum* (Shigao), *Pinellia ternata* (Thunb.) Breit. (Banxia), *Aster tataricus* Linn. f. (Ziwan), *Tussilago farfara* Linn. (Kuandong), *Belamcanda chinensis* (Linn.) Redouté (Shegan), *Asarum sieboldii* Miq. (Xixin), *Pericarpium Citri* Reticulatae (Chenpi), *Citrus aurantium* L. (Zhishi), *Zingiber officinale* Rosc. (Shengjiang), *Bupleurum chinensis* DC. (Chaihu), *Scutellaria baicalensis* Georgi (Huangqin), *Cinnamomum cassia* Presl (Guizhi), *Poria cocos* (Schw.) Wolf. (Fuling), *Alisma plantago-aquatica* Linn. (Zexie), *Polyporus umbellaru* (Pers.) Fr. (Zhuling), *Atractylodes macrocephala* Koidz. (Baizhu), *Dioscorea opposita* Thunb. (Shanyao), *Agastache rugosa* (Fisch. et Mey.) O. Ktze. (Huoxiang)	[41–43]
ShuangHuangLian oral liquid	*Lonicera japonica* Thunb. (Jinyinhua), *Scutellaria baicalensis* Georgi (Huangqin), *Forsythia suspensa* (Thunb.) Vahl. (Lianqiao)	[51]
Jinhua Qinggan granule	*Lonicera japonica* Thunb. (Jinyinhua), *Gypsum Fibrosum* (Shigao), *Ephedra sinica* Stapf (Mahuang), *Armeniacae Amarum* Semen (Kuxingren), *Scutellaria baicalensis* Georgi (Huangqin), *Forsythia suspensa* (Thunb.) Vahl. (Lianqiao), *Fritillaria thunbergii* Miq. (Zhebeimu), *Anemarrhena asphodeloides* Bunge (Zhimu), *Arctium lappa* L. (Niubangzi), *Artemisia carvifolia* Buch.-Ham. ex Roxb. (Qinghao), *Mentha haplocalyx* Briq. (Bohe), *Glycyrrhiza uralensis* Fisch. (Gancao)	[55, 56]
ReYanNing mixture	*Taraxacum mongolicum* Hand.-Mazz. (Pugongying), *Reynoutria japonica* Houtt. (Huzhang), *Sonchus brachyotus* DC. L. (Beibaijiang), *Scutellaria barbata* D. Don (Banzhilian)	[57]
Huopu Xialing decoction	*Agastache rugosa* (Fisch. et Mey.) O. Ktze. (Huoxiang), *Magnolia officinalis* Rehd. et Wils. (Houpu), *Pinellia ternata* (Thunb.) Breit. (Banxia), Semen Coicis Coix lachryma-jobiL.var.ma-yuen (Roman) stapf (Yiyiren), *Armeniacae Amarum* Semen (Kuxingren), *Poria cocos* (Schw.) Wolf. (Fuling), *Amomum kravanh* Pierre ex Gagnep. (Baidoukou), *Polyporus umbellaru* (Pers.) Fr. (Zhuling), *Alisma plantago-aquatica* Linn. (Zexie), *Tetrapanacis Medulla* (Tongcao), *Glycine max* (L.) Merr. (Dandouchi)	[66]
XiaoChaiHu decoction	*Bupleurum chinensis* DC. (Chaihu), *Pinellia ternata* (Thunb.) Breit. (Banxia), *Panax ginseng* C. A. Mey. (Renshen), *Glycyrrhiza uralensis* Fisch. (Gancao), *Scutellaria baicalensis* Georgi (Huangqin), *Zingiber officinale* Rosc. (Shengjiang), Ziziphus jujuba Mil1. Var·inermis (Bge·) Rehd. (Dazao)	[69]
MaXingShiGan decoction	*Ephedra sinica* Stapf (Mahuang), *Armeniacae Amarum* Semen (Kuxingren), *Glycyrrhiza uralensis* Fisch. (Gancao), *Gypsum Fibrosuum* (Shigao)	[77]
Yupingfeng prescription	*Astragalus membranaceus* (Fisch.) Bunge. (Huangqi), *Atractylodes macrocephala* Koidz. (Baizhu), *Saposhnikovia divaricata* (Trucz.) Schischk. (Fangfeng)	[69]

## Treatment strategies from Chinese herbal compounds

In the severe SARS-CoV-2 outbreak, there is a concerted global effort to develop new drugs to combat this major plague. The studies showed that COVID-19 would cause multi-organ damage, and brought enormous challenge to the treatment of COVID-19. Recently, emerging herbal compounds have been demonstrated helpful to improve COVID-19 by exerting antiviral activities, inhibiting cytokine storm, regulating immune functions, and so forth. The relevant research advances, especially the emerging clinical studies (Table 2), will provide comprehensive insights into the containment of the viral epidemic.

**Table 2:** The clinical/biological effects of Chinese herbal compounds for COVID-19 patients

Agents	Clinical/biological effects	Type of clinical study	References
Lianhua Qingwen prescription	Improved symptoms including fever, cough, muscle pain, fatigue and chest tightness	Single-center retrospective study	[12]
Shufeng Jiedu prescription	Time of defervescence ↓, disappear time of symptoms (dry cough, stuffy nose, runny nose, sore throat, fatigue, and diarrhea) ↓, time of SARS-CoV-2 turning negative ↓, clinical effectiveness ↑	Single-center retrospective study	[28]
	Improvement in pneumonia associated symptoms	Case report	[29]
	Time of defervescence ↓, white blood cell count ↑, lymphocyte percentage ↑, CRP ↓, IL-6 level ↓	Retrospective cohort study	[30]
Huoxiang Zhengqi formulation	Incidence of colds ↓, incidence of adverse events and adverse reactions ↓	Large cohort, prospective, randomized, and parallel-controlled study; randomized controlled trial	[35]
	Improved symptoms (nausea, vomiting and limb soreness), utilization rate of anti-infective drugs ↓, proportion of cases progressed to severe disease ↓	Randomized controlled study	[36]
Qingfei Paidu Tang	Risk of in-hospital mortality ↓	National retrospective registry study	[41]
	Improved creatine kinase, creatine kinase-myocardial band, lactate dehydrogenase, and blood urea nitrogen levels, CRP ↓	Single-center retrospective study	[42]
	Median length of hospital stay and nucleic acid negative conversion ↓, sputum disappearance time ↓, chest CT absorption rate ↑	Multi-center retrospective study	[43]
ShuangHuangLian oral liquid	Nucleic acid negative conversion rate ↑, inflammatory focus absorption ↑	Randomized, open-label, parallel-controlled, multi-center trial	[51]
Jinhua Qinggan granule	Duration of nucleic acid negative conversion ↓, pneumonia recovery time ↓	Single-center retrospective study	[55]
	Disappearance rate of clinical symptoms (fever, cough, fatigue, expectoration) ↑, anxiety score ↓	Prospective randomized controlled trial	[56]
ReYanNing mixture	Symptom scores (dry throat, cough, fatigue, chest tightness and headache) ↓, nucleic acid negative conversion rate ↑, median time to complete fever clearance ↓	Multi-center retrospective study	[57]
Huopu Xialing decoction	Improved liver function, viral nucleic acid and chest CT	Case report	[66]
XiaoChaiHu decoction	Disappearance rate of cough, sputum, shortness of breath, poor appetite and chills ↑, improvement rate of lung CT manifestations ↑	Single-center retrospective study	[69]
MaXingShiGan decoction	Effective rate ↑, antipyretic time ↓, cough disappearance time ↓	Prospective randomized controlled trial	[77]
Yupingfeng prescription	Shortened course of fever, cough and sputum, ameliorated symptoms such as fever, cough, sputum, painful pharynx, improved CRP and lung CT manifestations	Single-center retrospective study	[69]

*CRP* C-reactive protein, *CT* computerized tomography, *IL* interleukin, *SARS-CoV-2* Severe Acute Respiratory Syndrome Coronavirus 2

## Lianhua Qingwen prescription

Lianhua Qingwen is a commonly used Chinese medical preparation to treat viral influenza, especially in the fight against SARS in 2002–2003 in China [5, 11]. Wang et al*.* showed that 7 days after treatment with Lianhua Qingwen prescription (6 g, three times a day) in conjunction with conventional treatment for COVID-19 had a total effective rate of 92.73% accompanied by significantly reduced major symptoms including fever, cough, fatigue, and chest tightness [12]. The network pharmacology analysis confirmed that its effects was mainly focused on the biological processes such as the response to lipopolysaccharide (LPS), the molecular response to bacterial origin, the response to metal ions, and cell biological stimulation [12]. It may benefit the treatment of COVID-19 by the signaling pathways such as tumor necrosis factor (TNF), sarcoma-associated herpesvirus infection, interleukin (IL)-17, and human cytomegalovirus infection [12]. Other studies also showed that the effects of Lianhua Qingwen prescription was related to virus infection, inflammation, and immunity, moreover, its main active ingredients were verified by molecular docking with angiotensin-converting enzyme 2 (ACE2), the functional receptor of SARS-CoV-2, so as to have a therapeutic effect on COVID-19 [13, 14].

Lianhua Qingwen prescription also significantly inhibited SARS-CoV-2 replication in Vero E6 cells, markedly reduced the production of pro-inflammatory cytokines such as TNF-α, IL-6, chemokine (C-C motif) ligand 2 (CCL-2)/monocyte chemoattractant protein-1 (MCP-1), and chemokine (C-X-C motif) ligand 10 (CXCL-10)/interferon-inducible protein-10 (IP-10), and resulted in abnormal particle morphology of virion in cells [15]. Recent analysis indicated that Lianhua Qingwen prescription could act by regulating immune response, apoptosis, and virus infection [16]. Its active compounds beta-carotene, kaempferol, luteolin, naringenin, quercetin, and wogonin, could target RAC-alpha serine/threonine-protein kinase (Akt1), which is involved in lung injury, lung fibrogenesis, and virus infection, thereby helping eliminate virus infection with COVID-19 [16]. A meta-analysis involving 3793 subjects indicated that there was significant improvement of fever, fatigue, cough, and muscle pain in the Lianhua Qingwen group compared to the conventional drug group for treating common pneumonia and COVID-19 pneumonia [17].

Though emerging data showed its benefits on viral infection including SARS-CoV-2, the incidence of gastrointestinal reactions (diarrhea, abdominal distension, and gastrointestinal discomfort) was high as reported [18]. On April 14, 2020, the National Medical Products Administration (NMPA) of China approved modifying the specification of Lianhua Qingwen by adding a new indication for COVID-19 treatment [5]. However, its efficacy and safety should be confirmed by more clinical practices.

## Shufeng Jiedu prescription

Shufeng Jiedu prescription is composed of eight herbs [19]. An antibacterial testing suggested that Shufeng Jiedu prescription was a broad-spectrum antibacterial that superior to Lianhua Qingwen capsules with lowered mortality rate, increased average survival time, and increased lifespan of mice dying due to a Staphylococcus aureus or Streptococcus infection [20]. It also had a broad spectrum of antiviral effect and has a good therapeutic effect on pneumonia caused by parainfluenza virus [21, 22].

Shufeng Jiedu prescription also played a significant role in regulating immune function by significantly reducing the levels of IL-1α, IL-1β, IL-2, IL-4, IL-10, TNF-α, interferon (IFN)-α, and IFN-γ in a rat model of streptococcus pneumoniae-induced pneumonia [23]. Network pharmacology analysis revealed that it reduced the activity of nuclear factor kappa-B (NF-κB) via several signaling pathways, while its active ingredients quercetin, wogonin, and polydatin could bind directly to the main protease (Mpro) of SARS-CoV-2 [24]. Another study showed that Shufeng Jiedu prescription may prevent COVID-19 through its active ingredients likely quercetin, luteolin, wogonin, and kaempferol by targeting TNF, IL-10, IL-2, IL-6, STAT1, and CCL-2 [25]. It was also demonstrated that Shufeng Jiedu prescription may exert immunomodulatory and anti-inflammatory effects to control the current coronavirus, while RELA, MAPK1, MAPK14, CASP3, CASP8 were also the key target genes [26, 27].

Shufeng Jiedu prescription (2.08 g, three times per day) has been recently reported effective in the improvement of pneumonia associated symptoms when treating the patients of COVID-19, in combination with western medicine including lopinavir/ritonavir (Kaletra) and/or arbidol [28, 29]. Shufeng Jiedu prescription in combination with Arbidol and traditional Chinese and western allopathic medicine to treat common-type COVID-19, could improve its recovery time, and had better clinical effectiveness with higher white blood cell count and lymphocyte percentage, but lower C-reaction protein (CRP) and IL-6 levels [30]. It was also showed that Shufeng Jiedu prescription added to standard antiviral therapy, significantly reduced fatigue as well as cough days of COVID-19 compared to standard antiviral therapy alone, which was significantly more effective when used within the first 8 days after the onset of symptoms [24]. These evidence strongly suggested that Shufeng Jiedu prescription could be used as a complementary treatment to improve the efficacy of COVID-19 patients.

## Huoxiang Zhengqi formulation

Huoxiang Zhengqi formulation is a Chinese medicinal formula that mainly composed of ten kinds of herbs [19]. Modern pharmacological studies have found that Huoxiang Zhengqi formulation has antiviral, anti-inflammatory, and immunomodulatory activities [31, 32]. A systematic review has evaluated the efficacy and safety of Huoxiang Zhengqi formulation in treating gastrointestinal type symptoms [33]. The results showed that the efficacy of Huoxiang Zhengqi formulas is better than that of western medicine (formulations Paracetamol and Amantadine Hydrochloride or ribavirin) [33]. It was also better than conventional treatment alone (rehydration and antiviral therapy) when combining with conventional treatment [33]. Besides, it is more effective in improving the clinical symptoms (chills, fever, and bowel diarrhea) than western medicine treatment. Early application of Chaihu Droplet pill (1.05 g, twice daily) and Huoxiang Zhengqi formulation (5 g, twice daily) in treating SARS could alleviate the injury in lung of SARS patients and the neutrophil dependent inflammatory reaction, and reduce the dosage of glucocorticoid used [34].

Yan and colleagues showed that the preventive administration of Huoxiang Zhengqi Oral Liquid (oral before meals, 10 mL/time, 2 times/day, a course of 5 days) could effectively protect against respiratory symptoms, such as colds, on community residents in the case of COVID-19 [35]. Recent randomized controlled clinical trial indicated that Huoxiang Zhengqi dropping pills and Lianhua Qingwen formulation combined with western medicine had significant advantages in reducing the utilization rate of anti-infective drugs and improving the prognosis for COVID-19 patients [36]. Huoxiang Zhengqi formulation has been recommended in the treatment of COVID-19 by the National Health Commission (NHC) of China [37]. Further well-designed trials with large sample size are warranted to clarify its regulatory mechanisms and medication safety.

## Qingfei Paidu Tang

Qingfei Paidu Tang (or named Qingfei Detox Soup or Qingfei Paidu Decoction) is a mixture of twenty-one herbal components [38, 39]. Fan et al*.* showed that Qingfei Paidu Tang could be used to relieve internal and external pressure, and regulate triple energizer by promoting lung *Qi*, dispelling evil and detoxification, moistening and dampening, and purging heat by removing water, which is suitable for the pathogenesis of COVID-19 including cold, dryness, and dampness, and should effectively improve clinical symptoms [38]. Xu et al*.* reported that quercetin, luteolin, kaempferol, naringin, and isorhamnetin were the main active components of Qingfei Paidu Tang in the treatment of COVID-19 [40]. These agents could inhibit inflammatory reaction, regulate immune function, reduce lung injury and protect nerve function by regulating targets to achieve the purpose of treating COVID-19, such as mitogen-activated protein kinase 1 (MAPK1), MAPK3, MAPK8, MAPK14, IL-6, RELA proto-oncogene (RELA), and signal transducer and activator of transcription 1 (STAT1) [40].

Qingfei Paidu Tang treatment was associated with a lower risk of in-hospital mortality than those not receiving it, without extra risk of acute liver injury or kidney injury among hospitalized COVID-19 patients [41]. In mild and moderate COVID-19 patients, the combination of Qingfei Paidu Tang with western medicine demonstrated improved CRP, creatine kinase, creatine kinase-myocardial band, lactate dehydrogenase, and blood urea nitrogen levels, tended to mitigate the extent of multi-organ impairment, but neither mortality nor length of hospitalization was affected [42]. However, recent report showed that the single use of Qingfei Paidu Tang was more effective than the combination regimen with western medicine or other proprietary Chinese medicine [43]. It demonstrated that Qingfei Paidu Tang could significantly promote the improvement of nucleic acid negative conversion, chest CT, and sputum symptoms, and shortening the length of hospital stay than the combination therapy in light/common type of COVID-19 patients from five hospitals [43]. Another retrospective multicenter cohort study including 782 confirmed COVID-19 patients indicated that Qingfei Paidu Tang was associated with improved outcomes including faster recovery, shorter time to viral shedding, and less duration of hospital stay [44]. Further multicenter, prospective trials should be conducted to further confirm its clinical value.

## ShuangHuangLian oral liquid

ShuangHuangLian is a Chinese traditional patent medicine composing of three herbs [45]. ShuangHuangLian oral liquid combined with oseltamivir showed much higher effective rate and immunoglobulin A (IgA), IgM, and IgG levels than that of oseltamivir after the treatment of pediatric influenza [46]. Meanwhile, the remission time of patients with fever, cough, and general malaise was obviously shorter with ShuangHuangLian oral liquid treatment [46]. In vitro, either ShuangHuangLian oral liquid, the lyophilized powder of ShuangHuangLian for injection or their bioactive components dose-dependently inhibited 3-chymotrypsin-like protease (3CLpro) of SARS-CoV-2 and SARS-CoV-2 replication in Vero E6 cells [47]. In addition, two ingredients of ShuangHuangLian, baicalin, and baicalein, were the noncovalent, nonpeptidomimetic inhibitors of SARS-CoV-2 3CLpro and exhibited potent antiviral activities in a cell-based system [47].

Network pharmacology analysis showed that ShuangHuangLian oral liquid (the active formulations included quercetin, beta-sitosterol, neobaicalein) shared 28 targets with COVID-19, such as caspase-3, tumor protein p53 (TP53), MAPK8, IL-6, CCL-2, and the main pathways related to hepatitis b, TNF signaling pathway, pulmonary tuberculosis, whooping cough, salmonella infection, swine flu, as well as herpes simplex virus infection pathway [45]. Further evidence indicated that the chemical components of ShuangHuangLian had a good binding ability with 3CLMPro, ACE2, and the complex [48]. Besides, ShuangHuangLian exerted the effects of antivirus, anti-inflammatory, inhibition of oxidative stress and cell apoptosis, and may also be related to inhibit virus infection of host cells and interfere with virus replication and proliferation [48, 49]. In clinical, Ni et al*.* then reported three cases from a family case of COVID-19 treated with the combination of western medicine and ShuangHuangLian oral liquid (20 mL, twice daily), suggesting the expected therapeutic effects of ShuangHuangLian oral liquid on COVID-19 [50]. Patients with 14-day ShuangHuangLian oral liquid treatment had significantly higher rate in negative conversion of SARS-CoV-2 in nucleic acid swab tests with more absorption of inflammatory focus of pneumonia than the standard therapy alone [51]. It provides the theoretical basis and scientific basis for the treatment of COVID-19 with ShuangHuangLian oral liquid.

## Jinhua Qinggan granule

Jinhua Qinggan granule (JHG) composes of twelve herbs [52]. In a study of treating influenza of wind-heat affecting *Fei* syndrome, Li et al. divided the patients into the high dose JHG group (10 g each time), the low dose JHG group (5 g JHG + 5 g placebo each time), and the placebo control group (10 g placebo each time), and all medication was administered three times daily for 5 days [52]. The result demonstrated that the markedly effective rate of TCM symptoms and the recovery rate in the low dose JHG group was significantly higher than that of other groups both after 3 days and 5 days after treatment, respectively [52]. Then similar effects and safety were obtained in the low dose JHG group and the high dose JHG group, but the effects of the high dose JHG group was slightly poor in partial indicators, revealing the routinely low dose was the optimal dosage of JHG [52].

The “herbal medicine-compound-target” study elucidated that JHG was involved in TNF, phosphoinositide 3-kinase (PI3K)/Akt and hypoxia inducible factor (HIF)-1 signaling pathways which were related to lung injury protection, meanwhile, formononetin, stigmasterol, beta-sitosterol, anhydroicaritin and other key compounds of JHG have a certain degree of affinity with SARS-CoV-2 3CL hydrolase and ACE2 [53]. Recent network pharmacology study indicated that the biological processes involved in the key gene targets of JHG were cytokine activity, MAPK activity, chemokine activity, inflammatory response, immune response, while the signaling pathways in the treatment of COVID-19 included TNF, influenza A, HIF-1, NOD receptor, toll-like receptor (TLR), vascular endothelial growth factor (VEGF), MAPK, and T cell receptor signaling pathways [54].

In COVID-19 patients, JHG could effectively shorten the duration of nucleic acid detection and increase the absorption of pneumonia inflammatory exudate [55]. Compared with conventional treatment, the combination with JHG (6 g, three times daily for 5 days) significantly reduced the clinical symptoms of fever, cough, fatigue, expectoration, and relieved the anxiety of patients with mild COVID-19, but the incidence of adverse reactions in JHG group was significantly higher [56]. These data support that JHG should act as an effective agent in treating COVID-19, but its regulatory mechanism, effects, and safety are needed to be clarified with large sample data in the real world.

## ReYanNing Mixture

ReYanNing Mixture has good effects in the treatment of upper respiratory tract infection, cold, fever, acute pharyngitis, pneumonia, and many other respiratory diseases [57]. In a HCoV-229E infected mouse model, ReYanNing Mixture markedly reduced the lung index of coronavirus pneumonia mice, increased the percentage of CD8^+^ T lymphocytes, CD4^+^ T lymphocytes, total B lymphocytes, and reduced virus load as well as the levels of IFN-γ, TNF-α, IL-6, and IL-10 [58]. The in vivo study suggested obvious effects of ReYanNing Mixture in improving lung lesions, enhancing the autoimmune function, and reducing the inflammatory factors release, which provided laboratory evidence for its potential clinical benefits [58].

In addition, ReYanNing Mixture was reported to produce marked anti-inflammatory effects by regulating CD40LG, CXCL10, CXCL8, IL-10, IL-2, and IL-6, which was involved in IL-17 signaling pathway and cytokine-cytokine receptor interaction pathway by network topology analysis [59]. The molecular docking results revealed that its active compounds including apigenin, chrysin, and catechin, were identified with higher docking score rank against SARS-CoV-2 3CL protease, most of them were attributed to flavonoids [59]. Furthermore, the candidate targets including TNF, IFN-γ, TP53, CRP, and peroxisome proliferator-activated receptor γ (PPARγ), were identified among the bioactive ingredients of ReYanNing Mixture with effects on treating SARS-CoV-2 [60].

Recent study showed that the symptom scores such as dry throat, cough, fatigue, chest tightness and headache in COVID-19 patients was significant reduced with the treatment using ReYanNing Mixture (10–20 mL, 2–4 times per day) on the basis of chemical drug treatment compared with the chemical drug treatment only [57]. Besides, the median time to complete fever clearance with ReYanNing Mixture treatment was shorter, and the negative conversion rate of viral nucleic acid detection was higher [57]. These data revealed that ReYanNing Mixture may benefit COVID-19 by both anti-inflammatory and anti-virus actions, but the clinical evidence were far from sufficient.

## Huopu Xialing Decoction

Huopu Xialing Decoction consists of eleven herbs [61]. A few years ago, a small sample clinical trial had showed that Huopu Xialing Decoction has good clinical efficacy in treating *Pi-Wei* dampness-heat syndrome [62]. Recent study has also confirmed that the application of Huopu Xialing Decoction in the treatment of COVID-19 patients in Wuhan of China showed obvious clinical efficacy, and 697 targets were screened from TCM Systems Pharmacology Database, and 43 targets of which were related to COVID-19 and mainly involved in the IL-17 and NF-κB signal pathways [61].

A network pharmacology study showed that quercetin, luteolin, and baicalein were the main compounds of Huopu Xialing Decoction, which might regulate multiple signaling pathways targeting Akt1, MAPK1, cyclin D1 (CCND1), and caspase-8, thus played a therapeutic role on COVID-19 [63]. Meanwhile, other similar researches further demonstrated that the active components of Huopo Xialing Decoction had anti-inflammatory, immune regulation, anti-pulmonary fibrosis effects, and might protect against COVID-19 by regulating the biological processes such as blocking the protein synthesis of SARS-CoV-2 virus, preventing the virus from entering the host cells, and regulating TNF, MAPK, TLR, NOD-like receptor, and HIF-1 signaling pathways [64, 65]. Recent study showed that Huopo Xialing Decoction had obvious effects on improving symptoms, liver function, viral nucleic acid and chest CT in the treatment of COVID-19 patients with liver injury caused by other antiviral drugs [66]. Further clinical evidence in COVID-19 is still needed.

## XiaoChaiHu Decoction

XiaoChaiHu Decoction is a TCM compound preparation consisting of seven kinds of herbs [67]. Modern studies have shown that XiaoChaiHu Decoction can induce the activities of natural killer (NK) cells and T cells in liver tissues, and induce immune factors such as IL-1β, IL-2, and IFN-γ, so as to enhance the ability of virus clearance [19, 68]. A network pharmacology study showed that 140 active ingredients in XiaoChaiHu Decoction was for pneumonia treatment and immune regulation through 95 key targets such as IL-6, inducible nitric oxid synthase (NOS2), and estrogen receptor 1 (ESR1), involving TNF and IL-17 signaling pathways, and influenza A, of which 12 ingredients had direct anti-SARS-CoV-2 activity including baicalein, formononetin, quercetin [67]. The analysis result also indicated that the active ingredients in XiaoChaiHu Decoction treated COVID-19 by inhibiting SARS-CoV-2 activity, blocking SARS-CoV-2 invasion, inhibiting cytokine storm, and regulating immunity, but may not be effective for diseases such as alveolar inflammation.

A small sample clinical study proved that XiaoChaiHu Decoction and YuPingFeng prescription in combination with conventional therapy significantly shortened the course of fever, cough, and sputum in suspected cases of COVD-19, when compare to conventional therapy alone [69]. Besides, the combination treatment regimen also ameliorated the symptoms such as fever, cough, sputum, painful pharynx, shortness of breath, chills, and improved the CRP as well as lung computed tomography (CT) manifestations in patients [69]. Collectively, XiaoChaiHu Decoction may play a beneficial role in COVID-19 treatment, but need further randomized controlled studies to understand its exact clinical efficacy and safety.

## MaXingShiGan Decoction

MaXingShiGan Decoction consists of four kinds of herbs [70]. MaXingShiGan Decoction, the solution of heat and lung antiasthmatic prescription, is often used to treat respiratory diseases such as upper respiratory tract infection, acute bronchitis, acute attack of chronic bronchitis, and bronchial asthma in clinical [19]. Hsieh and partners observed that MaXingShiGan Decoction exhibited an EC(50) of 0.83 ± 0.41 mg/mL against influenza virus A/WSN/33 (H1N1), with broad-spectrum inhibitory activity against different strains of human influenza A viruses, including clinical oseltamivir-resistant isolates and an H1N1pdm strain [71]. Besides, MaXingShiGan Decoction abolished viral entry that regulated by PI3K/Akt signaling pathway. Zhong and collages showed that MaXingShiGan Decoction could improve H1N1 influenza A virus-induced acute lung injury (ALI) in mice, accompanied by decreased lung cell apoptosis and reduced the serum content of TNF-α [72]. In a rat model of lipopolysaccharides (LPS)-induced pneumonia, the thrombin and TLR signaling pathway were demonstrated to be essential pathways for MaXingShiGan Decoction mediated anti-inflammatory effects, while glycyrrhizic acid (one major compound in MXSG) inhibited TLR agonists induced IL-6 production in macrophage [73].

The network analysis of MaXingShiGan Decoction in severe COVID-19 predicted its main active components (quercetin, kaempferol, wogonin, naringenin, and isorhamnetin) with key targets including CCL-2, IL-1β, IL-4, IL-6, IL-10, MAPK1, MAPK3, MAPK8, TNF, caspase-3, Akt1, TP53, VEGFA, epidermal growth factor (EGF), and EGF receptor (EGFR) [74, 75]. A variety of its active ingredients could act on inflammatory responses (inflammatory cells, inflammatory cytokines, and their signaling pathways), immune responses, virus defense, humoral immunity, and mucosal innate immune response, and thus played a therapeutic role on COVID-19 [1, 75].

Treating infantile pneumonia with MaXingShiGan Decoction was reported superior to improve the disappearance time of lung moist rales, wheeze, cough, and fever than that without MaXingShiGan Decoction treatment [76]. Recent study demonstrated that modified MaXingShiGan Decoction combined with Sanren Decoction combined with conventional treatment (antiviral, immunomodulator, intestinal flora regulator, symptomatic treatment) had higher effective rate, and shorter time of antipyretic and cough disappearance [77]. The data revealed the potential effects of MaXingShiGan Decoction in COVID-19 therapy.

## YuPingFeng prescription

YuPingFeng prescription is a classical complex prescription composed of three herbs in a dry weight ratio of 3:1:1 [8]. Total glycoside and polysaccharides of YuPingFeng, the mixed extracts from *Yupingfeng*, were demonstrated to have immunoregulatory, anti-inflammatory, and anti-fibrotic activities [78–80]. Recent clinical study showed that YuPingFeng granules (5 g, three times per day) had a significantly lower exacerbation rate and reduced second exacerbation risk than the placebo treatment with a good safety profile during the prevention of acute exacerbation of chronic obstructive pulmonary disease (AECOPD) [81]. The meta-analyses also reported that YuPingFeng was effective to treat asthma in children, meanwhile, YuPingFeng combined with routine western medicine therapy for recurrent respiratory tract infection could improve the total effective rate and immune function with no serious adverse reaction [82, 83].

Huang et al. had recently proved that the core active compounds in YuPingFeng prescription could regulate multiple signal pathways by binding with ACE2 to ESR1, prostaglandin-endoperoxide synthase 2 (PTGS2) and other targets based on network pharmacology and molecular docking technology [84]. YuPingFeng prescription and XiaoChaiHu Decoction combined with routine treatment could improve the outcomes of suspected COVD-19 patients than routine treatment alone [69]. However, it was just a small sample of suspected cases with COVD-19. The benefits of YuPingFeng prescription as well as its extracts in COVID-19 deserve further in-depth studies and clinical attempts.

## Concluding remarks and future perspectives

The global public health crisis infected by SARS-CoV-2 has resulted in a fatality rate of 2% among infected patients [4]. To wipe out such plague epidemic, increasing efforts have turned to exploring the effective treatment regimens. However, the development of novel drugs is a systematic project, which is often a long and arduous process. The discovery of new actions with old drugs on the market becomes the principal way to explore the treatment of COVID-19. The fight against such epidemic provides a chance to test the role of TCM in the treatment of emerging infectious diseases. As a unique medical practice, Chinese herbal compounds have exerted its advantages in the treatment of COVID-19 [4, 24, 42]. The official report showed that 91.5% of confirmed COVID-19 cases received TCM (including oral Chinese herbal compounds) in China while the total effective rate was more than 90%, indicating the satisfactory therapeutic superiority with TCM in the treatment of COVID-19 [85]. Up to now, NHC has released the latest versions of Guidelines for Diagnosis and Treatment of COVID-19 [86]. Several Chinese herbal compounds that illustrated in this review, such as Huoxiang Zhengqi formulation, Lianhua Qingwen prescription, Shufeng Jiedu prescription, Jinhua Qinggan granule, and Qingfei Paidu Tang have been recommended for the treatment of COVID-19 by NHC on account of the disease stage and symptom differentiation [86].

**Fig. 1:**
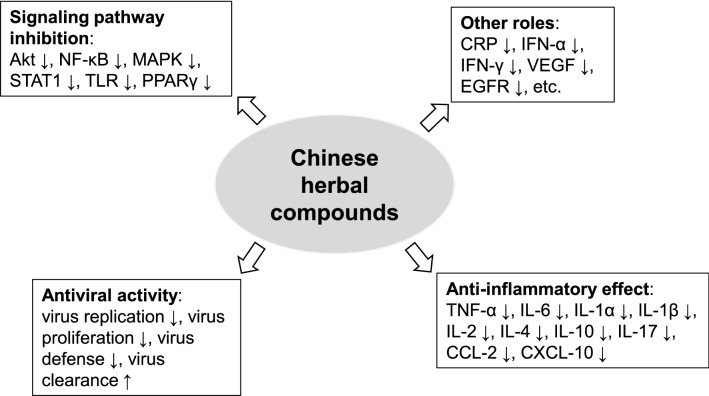
Potential pharmacological mechanisms of Chinese herbal compounds for COVID-19 therapy

The present study showed that the clinical effects of Chinese herbal compounds for COVID-19 treatment mainly included improved symptoms (fever, cough, muscle pain, fatigue) and lung CT manifestations, decreased risk of in-hospital mortality and adverse reactions, and increased inflammatory focus absorption as well as nucleic acid negative conversion rate. The updated evidence also demonstrated that the pharmacological mechanisms of Chinese herbal compounds for COVID-19 were mainly attributed to the down-regulated virus activity, inhibited cytokine storm, and enhanced immune function (Fig. 1). 3CLpro is critical for the replication and activity of virus, and thus may represent a promising therapeutic target for SARS-CoV-2 [87]. Here, the reports showed that following Chinese herbal compounds had the capacity to suppress the enzymatic activity of SARS-CoV-2 3CLpro: ShuangHuangLian oral liquid, Jinhua Qinggan granule, and ReYanNing Mixture [47, 48, 53, 59]. Interestingly, *Lonicera japonica* Thunb. (Jinyinhua), *Scutellaria baicalensis* Georgi (Huangqin), and *Forsythia suspensa* (Thunb.) Vahl. (Lianqiao) were the same herbal components of ShuangHuangLian oral liquid and Jinhua Qinggan granule. It was elucidated that the cellularentrance of SARS-CoV-2 was host receptor ACE2, and thus blockade of ACE2 holds the promise to prevent SARS-CoV-2 infection. In this study, we found that Lianhua Qingwen prescription, ShuangHuangLian oral liquid, Jinhua Qinggan granule, and YuPingFeng prescription may protect against SARS-CoV-2 infection by targeting ACE2 [13, 14, 48, 53, 84]. Similarly, the former three compounds contain herbal components of *Lonicera japonica* Thunb. (Jinyinhua) and *Forsythia suspensa* (Thunb.) Vahl. (Lianqiao). In-depth exploration of their active components on SARS-CoV-2 3CLpro and ACE2 may be beneficial for screening anti-COVID-19 agents.

In some patients, SARS-CoV2 infections will not turn into severe COVID-19 [88]. However, the systemic inflammatory response derived from lung injury caused by SARS-CoV-2 infection will result in cytokine storm and severe outcomes in COVID-19 [7, 88]. Therefore, anti-inflammatory agents presumably could decrease the severity and mortality rate of COVID-19 patients. Among the listed Chinese herbal compounds reviewed here, Shufeng Jiedu prescription, Qingfei Paidu Tang, ShuangHuangLian oral liquid, Jinhua Qinggan granule, Huopu Xialing Decoction, and MaXingShiGan Decoction were demonstrated to regulate MAPK signaling pathway [27, 40, 45, 54, 64, 74]. Meanwhile, ReYanNing Mixture, Huopu Xialing Decoction, and XiaoChaiHu Decoction had the activity to inhibit both IL-17 and TNF signaling pathways [59, 61, 67]. The inflammatory mediators like IL-6 and NF-κB were the other two major targets for most of the Chinese herbal compounds proposed in this study [25, 45, 61]. Chinese herbal compounds with the capacity to inhibit pro-inflammatory cytokine release may be harnessed to restrain multiple organ dysfunction and disease progression in the treatment of COVID-19.

However, there are potential study limitations that need to be addressed. First of all, the demonstrated efficacy of Chinese herbal compounds for COVID-19 were mostly from in vitro and in vivo studies, small-scale clinical trials, or the medication experience from the traditional use to COVID-19 therapy, and thus lack sufficient supporting evidence to some extent. Though the recent studies exerted therapeutic potential of Chinese herbal compounds for COVID-19, most of the clinical studies were retrospective studies or found to be poorly designed. It may lead to biases in evaluating the effects of Chinese herbal compounds. Therefore, further large-scale randomized controlled trials are still needed.

Secondly, growing studies speculated the potential mechanisms and benefits of Chinese herbal compounds for COVID-19 via network pharmacology-based analysis. It aims to reduce the time and cost of preclinical drug screening, and lead to more precise drugs and lower drug attrition rates. In fact, whether they protect against SARS-CoV-2 needs continuous experimental investigations as well as the feedback from serious clinical practice. In addition, the active molecules repeatedly found in different Chinese herbal compounds may not indicate a genuine result, but rather nonspecific interactions by specific molecules. Evaluating the importance of different targets will greatly facilitate the development of COVID-19 therapy.

Thirdly, Chinese herbal compounds in combination with western medicine had been reported effective to significantly improve the clinical symptoms and living quality during COVID-19 therapy. Nevertheless, the studies lacked the classification of disease severity and control group with Chinese herbal compounds treatment. Meanwhile, the clinical trials on the role of Chinese herbal compounds in COVID-19 mainly come from China, and none of these agents were approved by other countries till now. As searched in ClinicalTrials.gov (https://www.clinicaltrials.gov/), there are several ongoing clinical trials outside of China. Singapore designed a randomized controlled trial assessing the efficacy of Lianhua Qingwen as an adjuvant treatment in patients with mild symptoms of COVID-19 (ClinicalTrials.gov identifier: NCT04433013), but the recruitment status is “Not yet recruiting” up to now. Another study was to evaluate the effectiveness and safety of Jinhua Qinggan granules on mild-category patients of COVID-19 in Pakistani population (ClinicalTrials.gov identifier: NCT04723524), while the estimated study completion date was August 2021. We look forward to the evidence provided by these studies. Certainly, further well-designed multicenter trials from different countries are needed before an exact conclusion can be drawn. To work together against COVID-19, the exchange of experience in drug therapy should be strengthened, including the preferred use of Chinese herbal compounds that have been proven to be effective in multiple trials.

Fourthly, the dosages of Chinese herbal compounds for COVID-19 were based on previous published clinical data in other diseases. Besides, some of them are produced by different manufacturers, their quality standards may have differences. In order to obtain as accurate data as possible, the quality standards for drug production need to be harmonized. It is also necessary to investigate the effects of different doses of Chinese herbal compounds on different degrees of disease.

The last but not least, Chinese herbal compounds use may also bring adverse drug reactions, but the evidence is insufficient. Some Chinese herbal medicines contain renal toxins and mutagens, but their toxicological characteristics are still unclear. In addition, herbs used in TCM can mimic, amplify or counter the effects of traditional medicines. The complex components stand for multiple targets and pleiotropic effects, which may also bring more adverse drug reactions. Hence, the researches on the safety of Chinese herbal compounds should be carefully evaluated, especially from the real world data. In addition, it is of particular importance to avoid toxicity or interfere with the efficacy of routine treatment caused by herb-drug interaction. For example, it was not suitable to take both Lianhua Qingwen prescription and tonic herbs for COVID-19 cases at the same time. Due to the ethanol component contained in Huoxiang Zhengqi formulation, metronidazole, furazolidone, cefoperazone and sulbactam, and ceftriaxone as well as other drugs that could cause disulfiram-like reaction must be avoided in combination. It was also noteworthy that plenty of other TCM prescriptions that were crucial to the prevention and management of complicated diseases but not investigated yet, may shed light on more alternative natural compounds with the capacity to fight against COVID-19. In addition, standardized products of Chinese herbal compounds, rather than self-prepared formulations with no accepted standard of quality control, should be encouraged in the future study. The last but not least, it is recommended that clinical pharmacists can closely participate in COVID-19 treatment, such as prescription review and rational use of Chinese herbal compounds, to ensure the safety of drug use.

In conclusion, this study summarized the comprehensive and updated evidence of Chinese herbal compounds such as Lianhua Qingwen prescription, Shufeng Jiedu prescription, and Qingfei Paidu Tang for COVID-19 treatment. However, most of these studies were in vitro experiments or poorly designed retrospective trials, which may lead to potential biases in assessing the efficacy of Chinese herbal compounds in COVID-19. It is expected to further carry out randomized controlled trials in line with international standards and large-sample studies in the real world, to support the efficacy and safety of Chinese herbal compounds in the fight of COVID-19.

## Data Availability

The datasets are included within the article.

## Abbreviations

3CLpro: 3-Chymotrypsin-like protease
ACE2: Angiotensin-converting enzyme 2
Akt1: RAC-alpha serine/threonine-protein kinase
ALI: Acute lung injury
ARDS: Acute respiratory distress syndrome
CCL-2: Chemokine (C-C motif) ligand 2
CCND1: Cyclin D1
COVID-19: 2019 Coronavirus disease
CRP: C-reactive protein
CT: Computed tomography
CXCL-10: Chemokine (C-X-C motif) ligand 10
EGF: Epidermal growth factor
EGFR: Epidermal growth factor receptor
ESR1: Estrogen receptor 1
JHG: Jinhua Qinggan granule
LPS: Lipopolysaccharides
MAPK: Mitogen-activated protein kinase
MCP-1: Monocyte chemoattractant protein-1
NF-κB: Nuclear factor kappa-B
NHC: National Health Commission
NK: Natural killer
NMPA: National Medical Products Administration
NOS2: Inducible nitric oxid synthase
HIF: Hypoxia inducible factor
IFN: Interferon
IgA: Immunoglobulin A
IL: Interleukin
IP-10: Interferon-inducible protein-10
PI3K: Phosphoinositide 3-kinase
PPARγ: Peroxisome proliferator-activated receptor γ
PTGS2: Prostaglandin-endoperoxide synthase 2
RELA: RELA proto-oncogene
SARS: Severe Acute Respiratory Syndrome
SARS-CoV-2: Severe Acute Respiratory Syndrome Coronavirus 2
STAT1: Signal transducer and activator of transcription 1
TCM: Traditional Chinese medicine
TLR: Toll-like receptor
TNF: Tumor necrosis factor
TP53: Tumor protein p53
VEGF: Vascular endothelial growth factor
WHO: World Health Organization
